# A Novel Type of Blood Biomarker: Distinct Changes of Cytokine-Induced STAT Phosphorylation in Blood T Cells Between Colorectal Cancer Patients and Healthy Individuals

**DOI:** 10.3390/cancers11081157

**Published:** 2019-08-12

**Authors:** Jae Won Yun, Sejoon Lee, Hye Mi Kim, Sejong Chun, Edgar G. Engleman, Hee Cheol Kim, Eun-Suk Kang

**Affiliations:** 1 Department of Laboratory Medicine & Genetics, Samsung Medical Center, School of Medicine, Sungkyunkwan University, Seoul 06351, Korea; 2 Samsung Advanced Institute of Health Science and Technology, Sungkyunkwan University, Seoul 06351, Korea; 3 Samsung Genome Institute, Samsung Medical Center, Seoul 06351, Korea; 4 Department of Pathology, Bundang Hospital, Seoul National University, Seongnam-si, Gyeonggi-do 13620, Korea; 5 Samsung Biomedical Research Institute, Samsung Medical Center, Seoul 06351, Korea; 6 Department of Laboratory Medicine, Chonnam National University Medical School and Chonnam National University Hwasun Hospital, Gwangju 61469, Korea; 7 Department of Pathology, School of Medicine, Stanford University, Palo Alto, CA 94304-1204, USA; 8 Department of Surgery, Samsung Medical Center, School of Medicine, Sungkyunkwan University, Seoul 06351, Korea; 9 Stem Cell & Regenerative Medicine Institute, Samsung Medical Center, Seoul 06351, Korea

**Keywords:** STAT transcription factors, cytokines, tumor infiltrating lymphocytes, colorectal cancer, liquid biopsy

## Abstract

*Background*: Colorectal cancer (CRC) is one of the leading causes of cancer-related deaths worldwide. Although early diagnosis and treatment is the most successful strategy for improving patient survival, feasible and sensitive blood biomarkers for CRC screening remain elusive. *Methods*: Sixty-five CRC patients and thirty-three healthy individuals were enrolled. Peripheral blood (PB) and tumor tissues from CRC patients, and PB from healthy individuals were subjected to immunophenotyping and phospho-flow analysis of cytokine-induced phosphorylated STAT (CIPS). Logistic regression was used as a classifier that separates CRC patients from healthy individuals. *Results*: The proportion of regulatory T cells was increased in PB from CRC patients compared to PB from healthy individuals (*p* < 0.05). Interestingly, peripheral T cells share several cytokine-induced phosphorylated STAT (CIPS) signatures with T cells from CRC tumor-sites. Additionally, a classifier was made using two signatures distinct between T cells from CRC patients and T cells from healthy individuals. The AUCs (area under curves) of the classifier were 0.88 in initial cohort and 0.94 in the additional validation cohort. Overall AUC was 0.94 with sensitivity of 91% and specificity of 88%. *Conclusion*: This study highlights that immune cell signatures in peripheral blood could offer a new type of biomarker for CRC screening.

## 1. Introduction

Colorectal cancer (CRC) is the third most commonly diagnosed cancer in men and second in women [1,2]. Although early detection of CRC is important for reducing mortality [3], only 65% of the average-risk population in USA was reportedly compliant with CRC screening, including fecal occult blood testing (FOBT) and colonoscopy [4]. However, FOBT suffers from low sensitivity [5], and colonoscopy from increased risks of complication [6] and from poor compliance due to inconvenience.

For improved test convenience and feasibility, detecting the early stages of cancer, or following up on cancer relapse, has been attempted using various approaches to analyze biomarkers in peripheral blood. Specific strategies included signal detection of circulating tumor cells (CTC), circulating tumor DNA (ctDNA), or exosomes [7,8]. Recently, mRNA profiling of tumor-educated platelets (TEP) from cancer patients was suggested as a promising strategy for early detection, with good sensitivity and specificity [9].

Despite these advances, each strategy has its own obstacles and limitations to its clinical introduction. Methods used to detect CTC are of little use for tumor types having tumor cells with no tendency for extravasation [10]. In cases using exosomes, distinguishing between normal and tumor-cell-derived exosomes is difficult, as both show wide variation in size [11]. For cell-free DNA methods, good sensitivity can be achieved with high-throughput sequencing, especially in cases using a targeted approach. However, in an untargeted approach for tumors with an unidentified molecular profile, there is significant room for improved test sensitivity. In addition, there remain significant economic barriers, owing to testing costs [12]. Furthermore, these blood-based tests usually require blood stream invasion by cancer cells, which is generally indicative of later-stage cancers. The analysis of TEP in peripheral blood is a new method to screen for the existence of a tumor. However, complex modeling is needed because platelets only contain mRNA that originates from pre-mRNA, not from DNA, leading to weak tumor-educated signals [9]. 

Previously, Mortarini et al., reported an interesting observation, that tumor-infiltrating lymphocytes (TILs) and peripheral blood T cells had altered the IL-2-induced signal transducer, activator of transcription 1 (STAT1) and STAT5 phosphorylation in melanoma [13]. Although the underlying reasons remain unclear, those may be related to T-cell exhaustion, coupled with cytokine receptor dysregulation, or loss of effector cytokine secretion in the microenvironment of cancers or chronic pathogens [14]. Furthermore, decreased T-cell response to cytokines might be related to increased cytokine consumption owing to increased regulatory T cells with a high affinity for IL-2 receptors [13,15].

Expanding this observation to the CRC setting, we hypothesized that immune cells in peripheral blood from cancer patients share the cytokine-induced phosphorylated STAT (CIPS) signature of TILs, since immune cells circulate through blood vessels and lymphatic ducts. Hence, we investigated various cytokine-induced STAT phosphorylation signatures of peripheral blood mononuclear cells (PBMC) and TILs in CRC patients, using phospho-flow cytometry. We subsequently selected a combination of cytokines and STAT subtypes for which PBMC represents the signature of CRC TILs. We further evaluated the usefulness of the cancer-specific CIPS signatures in peripheral blood to discriminate CRC patients from healthy individuals.

## 2. Results

### 2.1. Patient Characteristics

Fifty patients were diagnosed with colorectal adenocarcinoma. Their characteristics are summarized in Table 1. The proportion of male and female cases were similar (46% versus 54%), and the average patient age was 67 years. Most cases presented with moderate differentiation in pathology (68%). Tumors from the right colon to rectum were most prevalent (74%) and numbers of cases for each of the tumor size, lymph node and metastasis (TNM) stages I, II, III and IV were six, 16, 18 and 10, respectively. Around half of all cases (44%) presented with positive lymphatic invasion.

### 2.2. Increased Proportion of Treg Cells in Th Cells of PBMCs and TILs from CRC Patients

**Figure 1:**
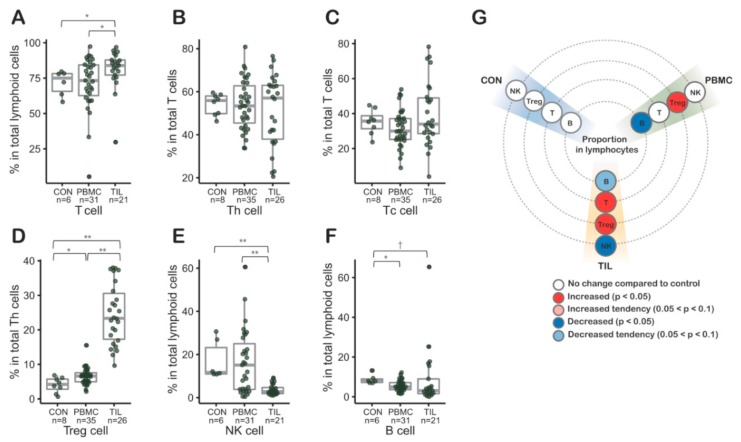
(**A**–**F**) Lymphocyte subsets in tumor infiltrating lymphocytes (TILs) and peripheral blood mononuclear cells (PBMCs) from colorectal patients (*n* = 50) by flow cytometry. (**D**) Regulatory T (Treg) cells of tumor infiltrating Th cells were proportionally significantly increased. In PBMCs from colorectal cancer (CRC) patients, the proportion of Treg cells in Th cells was also significantly increased. (**E**) The proportion of NK cells decreased in TILs. (**G**) Graphic visualization summarizing the lymphocyte subset results. CON, control peripheral blood mononuclear cells from healthy blood donors; PBMC, peripheral blood mononuclear cells from colorectal cancer patients; TILs, tumor-infiltrating lymphocytes from colorectal cancer patients.; Th, helper T cell; Tc, cytotoxic T cell; Treg, regulatory T cell; *, *p* values < 0.05; **, *p* values < 0.01 in *Mann-Whitney-Wilcoxon test*; middle line in box plot represents median and upper and lower sides are 1st and 3rd quantiles, respectively. Whiskers above and below the box plot represents values exceeding the 1st–3rd quantile range.

To investigate the immune cell distribution in PBMCs and TILs, we performed a lymphocyte subset analysis using flow cytometry (Table S1 and Section 4.3). Notably, the proportion of Treg cells in Th cells was significantly increased in both TILs (*p* < 0.01) and PBMCs from CRC patients (*p* < 0.05, Figure 1D,G). Compared to the control, the median increases of Treg proportion in Th of TIL and PBMC were 5.56 and 1.57 times, respectively (Figure 1D). In contrast, the proportions of CD4-positive T cells, representing Th cells, and CD8-positive T cells, representing Tc cells, were not increased in PBMCs from CRC patients. The proportion of NK cells was significantly decreased in TILs (*p* < 0.01, Figure 1) with median value ratio of TIL to control, 0.24.

### 2.3. CIPS of Peripheral Blood T Cells Represents CIPS of Tumor infiltrating T Cells

With an assumption that TILs could be affected by cytokine signals, especially JAK-STAT signals related with the immune response, PBMCs from CRC patients were analyzed to investigate how patients’ peripheral blood signatures were altered and whether they represented the status of TILs. Phospho-flow cytometry analysis was used to investigate how changes in CIPS signatures in TILs occurred. All analyses were performed in Th, Treg, and Tc cell subsets. We analyzed the CIPS signatures of IL-2, IL-6 and IL-10 (See Materials and Methods). IL-2 is important for maintenance of T cells, including Treg cells, and activates the JAK-STAT pathway through induction of STAT5 phosphorylation [13]. IL-6 contributes to the inflammatory responses through phosphorylation of STAT1 and STAT3 [16]. IL-10 stimulates STAT3 phosphorylation and stimulates the immunosuppressive environment [17,18]. Hence, we selected combinations of theses cytokines and STATs, while taking into account specimen amount and availability (Table S2).

**Figure 2:**
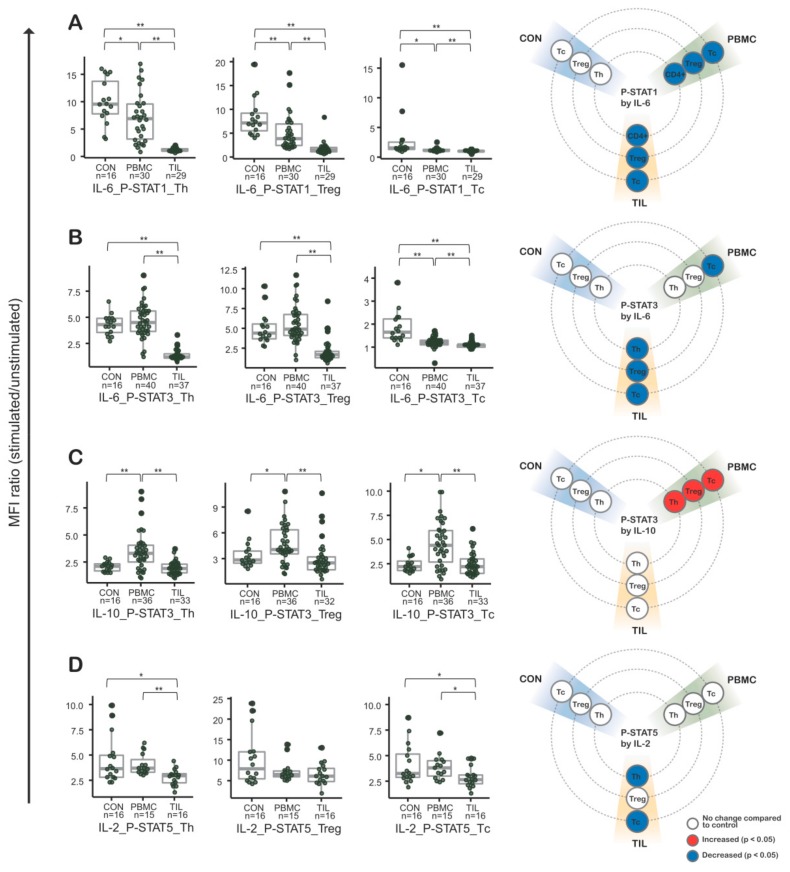
Cytokine induced phosphorylated STAT (CIPS) signatures in peripheral blood mononuclear cells (PBMCs) and tumor infiltrating lymphocytes (TILs) from colorectal cancer (CRC) patients (n = 50). Phospho-flow cytometry was performed in combination for IL-6 and STAT1 (**A**), IL-6 and STAT3 (**B**), IL-10 and STAT10 (**C**), and IL-2 and STAT5 (**D**). In the first three plots (from the left), CIPS signatures were tested in helper T cells, regulatory T cells, and cytotoxic T cells. To the far right is a visualization summarizing each CIPS signature. CON, control peripheral blood mononuclear cells from healthy blood donors; PBMC, peripheral blood mononuclear cells from colorectal cancer patients; TILs, tumor-infiltrating lymphocytes from colorectal cancer patients; CRC, colorectal cancer.; *, *p*-values < 0.05; **, *p*-values < 0.01 in Mann-Whitney-Wilcoxon test; middle line in box plot represents median and upper and lower sides are 1st and 3rd quantiles, respectively. Whiskers above and below the box plot represents values exceeding the 1st–3rd quantile range.

Intriguingly, IL-6-induced p-STAT1 (phosphorylated STAT1) and p-STAT3 were markedly decreased in TILs (Figure 2A,B). Additionally, PBMC CIPS signatures in CRC patients partially represented CIPS signatures in TILs. That phenomenon was observed in IL-6-induced p-STAT1 of Th, Treg, and Tc cells, as well as in IL-6-induced p-STAT3 of Tc cells. IL-10-induced p-STAT3 was similar in both TILs and control cells (Figure 2C), but significantly increased in patients’ PBMCs compared to TILs and control cases. Regarding IL-2 induced p-STAT5, there was significant decrease in Th and Tc of TILs compared to those of control or patients’ PMBCs (Figure 2D). But there was no definite difference between T cells of patients’ PBMCs and control.

Overall, those results indicate that stimulation from certain cytokines significantly alters downstream intracellular CIPS not only in TILs but also in the PBMCs from CRC patients.

### 2.4. CIPS of Peripheral Blood T Cells Co-Cultured with Colon Cancer Cells

**Figure 3:**
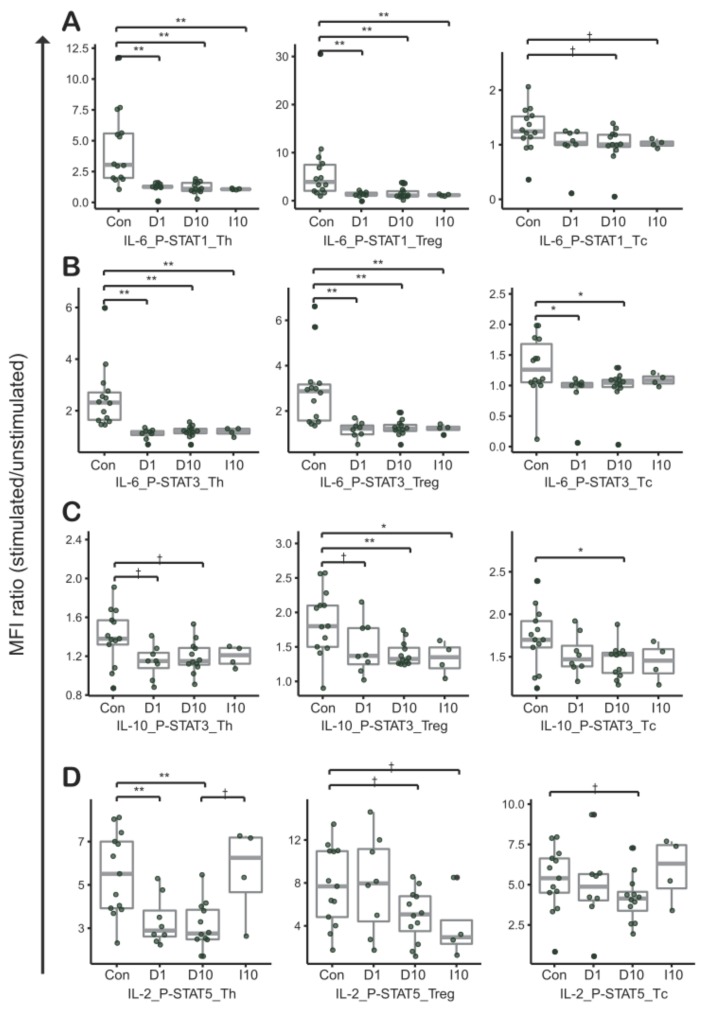
Cytokine induced phosphorylated STAT signatures between peripheral blood mononuclear cells (PBMCs) and PBMCs co-cultured with the HCT116 cancer cell line. Phospho-flow cytometry was performed in combination for (**A**) IL-6 and STAT1, (**B**) IL-6 and STAT3, (**C**) IL-10 and STAT10, and (**D**) IL-2 and STAT5. The overall pattern between control cells and PBMCs co-cultured with HCT116 cells is similar to the pattern between control cells and TILs in Figure 2. “Con” represents control (T cells from PBMCs of healthy donors, *N* = 14). “D1” represents T cells from PBMCs co-cultured in direct contact with HCT116 cells (1:1, *N* = 8). “D10” represents T cells from PBMCs co-cultured in direct contact with HCT116 cells (1:10, *N* = 12). “I10” represents T cells from PBMCs co-cultured in indirect contact with HCT116 cells (1:10, *N* = 4).; †, *p*-values < 0.1; *, *p*-values < 0.05; **, *p*-values < 0.01 in Mann-Whitney-Wilcoxon test; middle line in box plot represents median and upper and lower sides are 1st and 3rd quantiles, respectively. Whiskers above and below the box plot represents values exceeding the 1st–3rd quantile range.

To investigate the reproducibility of CIPS signatures of TILs and intravascular PBMCs adjacent to tumor in vitro, we conducted phospho-flow cytometry analyses of normal PBMCs co-cultured in direct or indirect contact with a colorectal cancer cell line, HCT116 (Table S3). For most combinations, results for the in vitro study were in agreement with results for T cells in TILs (Figure 2 and Figure 3). Particularly, CIPS signatures in directly co-cultured PBMCs were similar to the signatures for TILs in IL-6-induced p-STAT1 of Th and Treg cells, IL-6-induced p-STAT3 of Th, Treg, and Tc cells, and IL-2-induced p-STAT5 of Th cells (D1 and D10 in Figure 3A,B,D).

There was no distinct difference in CIPS signatures between T cells from PBMCs and HCT116 cells co-cultured in direct contact at ratios of 1:1 and 1:10 (D1 and D10 in Figure 3A–D). Compared to directly cultured T cells, a similar phenomenon was observed in T cells that were indirectly cultured (D10 and I10 in Figure 3A–C), except for the IL-2-induced p-STAT5 signature (D10 and I10 in Figure 3D). Although statistically not certain, due to a limited number of tested replicates, the increase of IL-2 induced p-STAT5 in I10 suggests cross-talk between T cells and cancer cells, through direct contact. That is important in IL-2-induced p-STAT5 signature alteration of Th and Tc cells (D10 and I10 in Figure 3D).

In summary, the results imply that CIPS signatures in T cells are reproducibly altered through crosstalk between T cells and CRC cells.

### 2.5. Distinct CIPS Signatures of Peripheral Blood T Cells from CRC Patients

**Figure 4:**
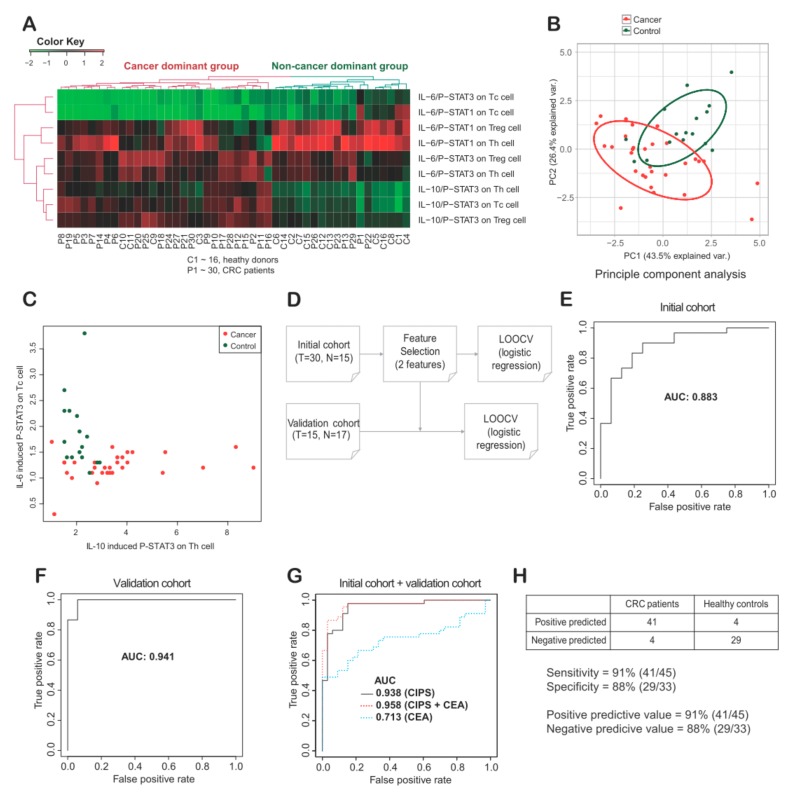
(**A**) Unsupervised cluster analysis among cytokine induced p-STAT (CIPS) signatures of peripheral blood mononuclear cells (PBMCs) from thirty colorectal cancer (CRC) patients and sixteen healthy donors. Signatures were divided into a cancer dominant group (*N* = 28) and a non-cancer dominant group (*N* = 18). (**B**) Principal component (PC) analysis showed PC1 and PC2 explains 43.5% and 26.4% of variance, respectively. (**C**) Two features with the largest Mean Decrease Gini were selected and plotted. (**D**) With the two features based on MeanDecreaseGini (see Materials and Methods), a simple logistic regression model was generated to distinguish CRC patients from healthy donors, and leave-one-out cross validation (LOOCV) was performed. (**E**–**G**) A receiver operating characteristic (ROC) curve was plotted for inferring the area under curve (AUC) value in training cohort, validation cohort and merged cohort. AUC values were provided in CIPS, CEA and CIPS plus CEA (**G**). (**H**) Sensitivity, specificity, positive predicted value and negative predictive value were described as 91%, 88%, 91% and 88%, respectively. Th, helper T cells; Treg, regulatory T cells; Tc, cytotoxic T cells.

PBMCs from 30 CRC patients and 16 healthy donors, with full CIPS signatures, were selected for cluster analysis. Two major groups were found to form clusters (Figure 4A), with the first major group consisting mainly of CRC patients (23/27; 85%), and the second major group consisting mainly of healthy donors (12/18; 67%). Between these two major groups, there were distinct differences in the IL-10-induced p-STAT3 signatures on Th, Treg, and Tc cells. A principal component (PC) analysis was subsequently performed using the same data set. PC1 and PC2 respectively showed 43.5% and 26.4% of variation that could be explained (Figure 4B). In PC1, IL-10-induced p-STAT3 on Th, Treg, and Tc cells was found to be the highest contributor. In PC2, IL-6-induced p-STAT1 on Th and Treg cells, as well as IL-6-induced p-STAT3 on Tc cells, were found to be the highest contributors.

To investigate the potential clinical utility of CIPS signatures as biomarkers for CRC, we selected specific CIPS signature features, made a simple statistical model, and validated the model using leave-one-out cross validation (LOOCV) (Figure S1A,B and Section 4.6). Two distinct CIPS features, IL-6-induced p-STAT3 on Tc cells and IL-10-induced p-STAT3 on Th cells, were selected based on mean decrease in Gini (MDG) values (Figure 4C). To minimize overfitting, a very simple model with multivariate logistic regression based on two features was inferred (Figure 4D). In the initial cohort, we performed LOOCV and calculated the area under curve (AUC) to be 0.88 (Figure 4E). Next, we performed the same analysis using peripheral blood (PB) samples from additionally enrolled 15 CRC patients and 17 healthy individuals, and the AUC was 0.941 (Figure 4F). Overall the AUC using all samples was 0.938 (Figure 4G). Additionally, using training data (initial data, *N* = 46), we made a logistic regression model and applied the model for validation data (*N* = 32). The accuracy and the AUC were 0.97 and 0.98, respectively. The results are similar with LOOCV, although the sample number is limited. The sensitivity and specificity were 91% and 88%, respectively (Figure 4H). We also assessed the effect on diagnostic performance by adding carcinoembryonic antigen (CEA), a clinically used blood biomarker for monitoring the clinical course of CRC. We have calculated AUC by adding CEA to the two CIPS features and interestingly, the AUC was increased to 0.958 (Figure 4G). The final multivariate logistic regression model is:

Y = −7.840 × X_1_ + 1.144 × X_2_ + 8.388 (1)

where, X_1_ is the MFI ratio of p-STAT3 before and after IL-6 stimulation of Tc cells, and X_2_ is the MFI ratio of p-STAT3 before and after IL-10 stimulation of Th cells.

## 3. Discussion

**Figure 5:**
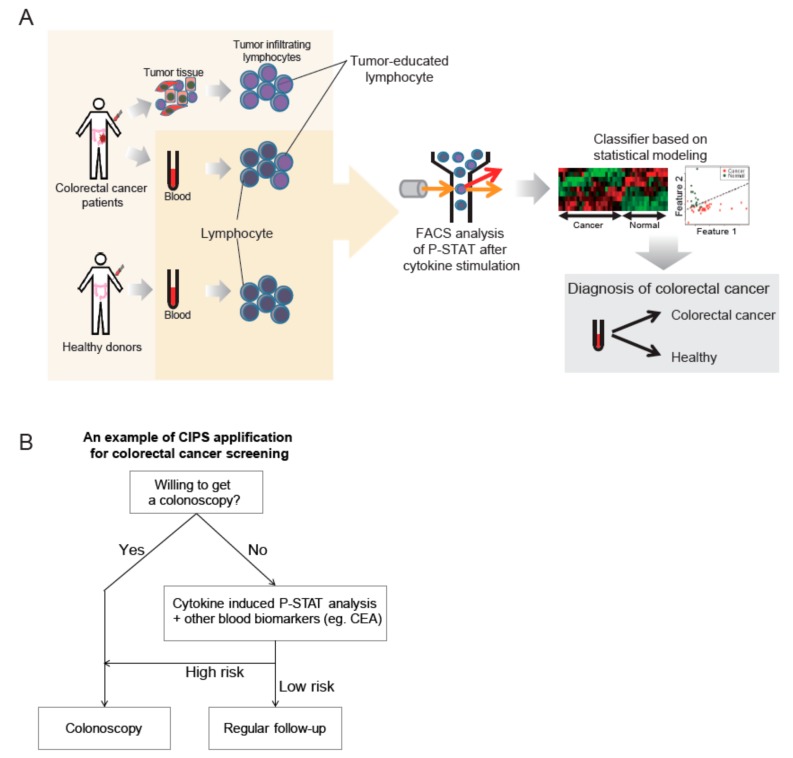
(**A**) Graphical overview of a novel type of biomarker, the cytokine induced p-STAT (CIPS) signature. CIPS signatures are different in peripheral blood T cells when comparing colorectal cancer (CRC) patients and healthy donors. After phospho-flow cytometry analysis was performed, a simple classifier using multivariate logistic regression was validated by leave-one-out cross validation. Final performance was tested using the receiver operating characteristic (ROC) curve and the area under curve (AUC) values. (**B**). For an example of bedside utility of this test, a modified screening strategy for CRC could be designed for individuals unwilling to have a colonoscopy in the clinical setting. In combination with other biomarkers from different underlying biological sources, improvement in test performance is to be expected for cancer screening by non-invasive liquid biopsy; especially for individuals that are unwilling to get a colonoscopy when not presenting with any CRC risk factors.

In the present study, peripheral blood T cells from CRC patients were shown to have CIPS signatures that could be distinguished from peripheral T cells of healthy individuals. Furthermore, most CIPS signatures for peripheral blood T cells correlated with those of T cells in TILs. In addition, the CIPS signatures were reproducible in PBMCs co-cultured with colorectal cancer cells. This suggests the clinical utility of CIPS signatures in peripheral blood T cells as biomarkers to distinguish CRC patients from healthy individuals. CIPS signatures differ from other blood biomarkers, such as CEA or ctDNA that originate from tumor cells, since CIPS signatures arise from the direct or indirect cross-talk between circulating immune cells and cancer cells. In this study, we believe that CIPS in peripheral blood is a valuable type of biomarker to determine cancer or non-cancer status, while a robust validation with blood samples from a large number of individuals, with various medical conditions is required to get this test to the bedside. For an example of the bedside utility of this test, a modified screening strategy for CRC could be designed for individuals unwilling to have a colonoscopy in the clinical setting (Figure 5). 

Biologically, exhaustion of T cells in the tumor microenvironment could possibly account for altered CIPS signatures in T cells. Under conditions of antigen-persistence by either infection or cancer, loss of T-cell function could occur in a progressive manner through T-cell exhaustion [14]. In a chronic state of both immune suppression and antigen persistence, exhausted T cells have been shown to have the following characteristics: (i) Impaired function with a decrease in cytokine production, cytotoxicity, proliferation and self-renewal; (ii) cytokine receptor down-regulation, including IL-2Rβ and IL-6R; (iii) up-regulation of inhibitory receptors; e.g., PD-1 and CTLA-4; (iv) altered transcription factor expression; e.g., Blimp-1, BATF, T-bet and Eomes; and (v) altered signaling molecule regulation; e.g., NFAT or SOCS3, which is related with IL-6 responsiveness [14,19,20].

Increased Treg cells containing high affinity cytokine receptors could be related with increased cytokine consumption [13,15], and our finding that Treg cells increased in CRC patients supports this possibility. However, the detailed mechanism of CIPS alteration in each type of immune cell was not fully investigated till now. Previously, Zhou et al. reported that the effect of IL-6 on Th cells depends on STAT3, which is related to Th17 cell differentiation [21]. In another study, hyper-IL-6 treatment, in combination with anti-CD3/CD28-coated microbeads, induced p-STAT3 in T cells, indicating that hyper-IL-6 initiates an as-yet-undefined signaling cascade related to effector T-cell differentiation [19]. IL-10 is an immunosuppressive cytokine produced by variable immune cells, especially Treg cells [22], which are rich in TILs in our study. In a melanoma study, Sun et al. reported that CD8+ TILs present in tumor site upregulate IL-10R and PD-1, and that PBMCs of melanoma patients produce IL-10, which limits the proliferation and survival of IL10R+ tumor antigen-specific CD8+ T cells [23]. In our study, interestingly, p-STAT3 induction sensitive to IL-10 stimulation was observed in T cells of PBMCs (Figure 2C), while the IL-10-induced p-STAT3 signature was retained in T cells of TILs. Based on the observation, peripheral blood T cells of CRC patients could induce p-STAT3 more sensitively to IL-10 stimulation, indicating IL-10 hypersensitivity in peripheral blood T cells of CRC patients. Further investigation of the underlying biological mechanisms behind this observation is required.

## 4. Materials and Methods

### 4.1. Study Participants

Total of 50 Korean patients diagnosed with CRC and who underwent colectomy at the Samsung Medical Center were included. Forty patients were subjected to the cytokine-induced p-STAT (CIPS) analysis for PBMCs and/or TILs. Thirty-eight patients were subjected to the lymphocyte subset analysis in PBMC and/or TILs. In some patients, all profiling could not be performed due to lack of specimens. (Table S1). Total 30 healthy donors were included as the controls provided by the Korea Gynecologic Cancer Bank. PBMCs were used for either subset analysis (*N* = 8) or initial CIPS analysis (*N* = 16) and for in vitro co-culture study (*N* = 14). For the validation cohort, fifteen CRC patients and seventeen healthy individuals were additionally enrolled. Fresh CRC tissue and peripheral blood were obtained from patients on the day of surgery. We reviewed patient medical records for clinical characteristics including age, gender, gross type, differentiation, lymphatic invasion, venous invasion, perineural invasion, and the Union for International Cancer Control (UICC) tumor-node-metastasis (TNM) classification. Histologic types except adenocarcinoma were excluded. This study was reviewed and approved by the Institution Review Board. All subjects submitted written informed consent.

### 4.2. Sample Collection and Preprocessing

Tumor samples were taken from each surgically removed specimen immediately following removal from the patient. Single-cell suspensions were prepared, and tumor tissues were mechanically dissected and suspended in phosphate-buffered saline (PBS). Enzymatic treatment was not used to dissociate tumors because it affected CIPS expression. Cell suspensions were subsequently passed through a sterile 40 μm nylon filter (BD Falcon, Heidelberg, Germany). Single cells were pelleted and suspended in RPMI 1640 (Life Technologies, Grand Island, NY, USA) containing 10% fetal bovine serum (FBS, Life Technologies), penicillin-streptomycin (WelGENE, Daegu, Korea), and L-glutamine (WelGENE). PBMCs were isolated by Ficoll (GE Healthcare, Piscataway, NJ, USA) density gradient centrifugation of whole blood from healthy donors and CRC patients. Cells were counted following trypan blue staining and the concentration adjusted to 0.5–1 × 10^6^ viable cells/tube.

### 4.3. Immunophenotyping

PBMC surface expression analysis was performed by multicolor staining. PBMCs or single cells isolated from tumor tissue were distributed as 100 µL cell suspension aliquots (10^6^ cells) in FACS tubes, stained with specific monoclonal antibodies, and fixed/permeabilized (eBiosciences, San Diego, CA, USA) for intracellular staining. Cytotoxic, helper, and regulatory T-cell analysis was performed by staining cells with monoclonal antibodies against CD45 (clone 2D1), CD3 (clone SK7), CD4 (clone SK3), CD8 (clone RPA-T8), CD25 (clone 2A3), and FOXP3 (clone PCH101). The myeloid cell fraction was detected by staining with monoclonal antibodies against CD45, CD3, CD19 (clone HIB19), CD56 (clone CMSSB), CD11b (clone ICRF44) and CD11c (clone 3.9). NK cells were evaluated by staining with monoclonal antibodies against CD45, CD3, CD56 and iNKT (Vα24-Jα11). All antibodies were manufactured by eBioscience or BD Biosciences. Cells were analyzed by FACSCanto II using FACSDiva v6.1.3 (BD Biosciences, San Jose, CA, USA). The information to define the combination of specific markers for each immune cell type was provided in Table S4.

### 4.4. STAT Phosphorylation Before and After Cytokine Stimulation

To observe STAT phosphorylation, single-cell suspensions of CRC tumor tissue and PBMC were stimulated with 10 ng IL-2, IL-6, and IL-10 for 15 min at 37 °C. For intracellular phospho-protein staining for flow cytometry, stimulated and unstimulated cells were fixed with a final concentration of 1.5% paraformaldehyde in culture medium for 10 min and subsequently pelleted. Thoroughly resuspended cells were permeabilized in 1 mL ice-cold MeOH per 10^6^ cells and stored at −70 °C for approximately two weeks before staining. Cells were washed three times with PBS containing 0.5% BSA, and resuspended in staining media at 0.5–1 × 10^6^ cells per 100 μL. Anti-phospho-STAT-1 (pY701), anti-phospho-STAT-3 (pY705), and anti-phospho-STAT-5 (pY694) antibodies (BD Biosciences, San Diego, CA, USA), as well as monoclonal antibodies against lineage markers, were added and incubated in the dark for 30 min. Cells were subsequently washed with staining media and pelleted. Finally, samples were resuspended in 200 μL staining media and analyzed by FACSCanto II using FACSDiva v6.1.3 (BD Biosciences, San Diego, CA, USA).

### 4.5. In Vitro Co-Culture of PBMCs with a Cancer Cell Line

Fresh PBMCs from healthy donors and a human colorectal carcinoma cell line, HCT116 (ATCC CCL-247), were co-cultured overnight at 1:1 or 1:10 ratios. Cells were either cultured in direct contact or separately in transwell plates, with or without the addition of IL-2 (20 ng/mL), IL-6 (10 ng/mL), or IL-10 (10 ng/mL). Co-cultured PBMCs in direct contact with HCT116 mimicked the TILs. Cultured PBMCs in transwell plates were allowed to exchange soluble factors through the membrane but without direct contact with HCT116. Those PBMCs mimicked circulating immune cells in vessels adjacent to tumor. Eight PBMCs were co-cultured with HCT116 in direct contact in a 1:1 ratio. Twelve PBMCs were co-cultured with HCT116 in direct contact in 1:10 ratio. Four were co-cultured with HCT116 in indirect contact in 1:10 ratio (Table S3). Co-cultures were maintained for 24 h in RPMI 1640 (Life Technologies, Grand Island, NY, USA) containing 10% FBS. Thereafter, cells were subjected to phospho-flow cytometric analysis as described above for single-cell suspensions of tumor tissue or PBMCs. 

### 4.6. Cluster Analysis, Classification Using Logistic Regression, and Validation

For cluster analysis, the hierarchical method was used. A heatmap was drawn using the R package, gplots [24], and a principle component analysis performed to identify the highest contributing factors. To select the most important features that could discriminate between CRC patients and healthy controls, we calculated the feature importance value of the mean decrease in Gini (MDG) using the randomForest package in R [25]. The Gini impurity index was calculated as follows:

(2) $$G=\sum_{i=1}^{n}p_{i}\left(1-p_{i}\right)$$

where *n* is the number of classes in the target variable and $p_{i}$ is the ratio of this class. 

After ranking the important features using MDG values for all CIPS features, we investigated the correlation among these features using the Pearson correlation test. To reduce redundant features, we excluded features showing high correlation values (*R* > 0.7) compared with the higher-ranked features. With the top two ranked features selected (MDG > 3), we developed a logistic regression model, which was validated using leave-one-out cross validation (LOOCV). The receiver operating characteristic (ROC) curve was plotted, and the area under curve (AUC) was calculated using the ROCR package [26].

### 4.7. Statistical Analyses

All CIPS basal level ratios were calculated by dividing the median fluorescence intensity (MFI) of stimulated cells by the MFI of unstimulated cells. The Mann-Whitney-Wilcoxon test was used to determine the statistical differences of the p-STAT ratios between patients’ PBMCs and controls, between TILs and controls, between patients’ PBMCs and TILs, and between PBMCs co-cultured with HCT116 cells and controls. All statistical analyses were performed using R [27]. *p*-values < 0.05 were considered statically significant.

## 5. Conclusions

In conclusion, CIPS signatures offer a novel type of biomarkers for use in CRC diagnosing tests employing a liquid biopsy. Although we believe that this novel type of blood biomarker provides a good chance in the field of CRC screen, robust validation using large number of clinical specimen would be needed for clinical utility.

## Figures and Tables

**Table 1:** Characteristics of colorectal cancer patients initially enrolled.

Features		Case Number (Proportion)
**Age (Mean and SD)**		67 ± 9
**Gender**	Female	23 (46%)
	Male	27 (54%)
**Differentiation**	Well	12 (24%)
	Moderately	34 (68%)
	Poorly	4 (8%)
**Location**	Ascending colon	8 (16%)
	Descending colon	1 (2%)
	Rectosigmoid junction	4 (8%)
	Sigmoid colon	14 (28%)
	Transverse colon	4 (8%)
	Rectum	18 (36%)
	Cecum	1 (2%)
**Side**	Left	13 (26%)
	Right	37 (74%)
**TNM stage**	I	6 (12%)
	II	16 (32%)
	IIIa	0 (0%)
	IIIb	9 (18%)
	IIIc	9 (18%)
	IVa	6 (12%)
	IVb	4 (8%)
**Invasion**	Lymphatic	22 (44%)
	Venous	14 (28%)
	Perineural	9 (18%)
**Tumor budding**	Negative	24 (48%)
	Positive (5–9)	19 (38%)
	Positive (≥10)	7 (14%)

Abbreviations: PBMC, peripheral blood mononuclear cell; TNM, tumor size, lymph node and metastasis.

## Supplementary Materials

The following are available online at https://www.mdpi.com/2072-6694/11/8/1157/s1, Figure S1, Table S1: Data of cytokine induced p-STAT signature in peripheral blood cells, directly or indirectly cultured with HCT116.
