# Supplementary material for: A Novel Type of Blood Biomarker: Distinct Changes of Cytokine-Induced STAT Phosphorylation in Blood T Cells Between Colorectal Cancer Patients and Healthy Individuals
Source: Cancers (Basel). 2019 Aug 12;11(8):1157. doi: 10.3390/cancers11081157 (PMC6721561; doi:10.3390/cancers11081157)
Supplement: Supplementary file 1 [file cancers-11-01157-s001.zip › cancers-524079-Supplementary Figures.pdf]

A

Features	MeanDecreaseGini
IL-6 induced P-STAT3 on CD8+ T	3.588
IL-10 induced P-STAT3 on CD4+ T	3.539
IL-10 induced P-STAT3 on CD8+ T	3.385
IL-6 induced P-STAT1 on regulatory T	2.387
IL-6 induced P-STAT1 on CD8+ T	2.032
IL-10 induced P-STAT3 on regulatory T	1.701
IL-6 induced P-STAT1 on CD4+ T	1.554
IL-6 induced P-STAT3 on CD4+ T	0.909
IL-6 induced P-STAT3 on regulatory T	0.85

B

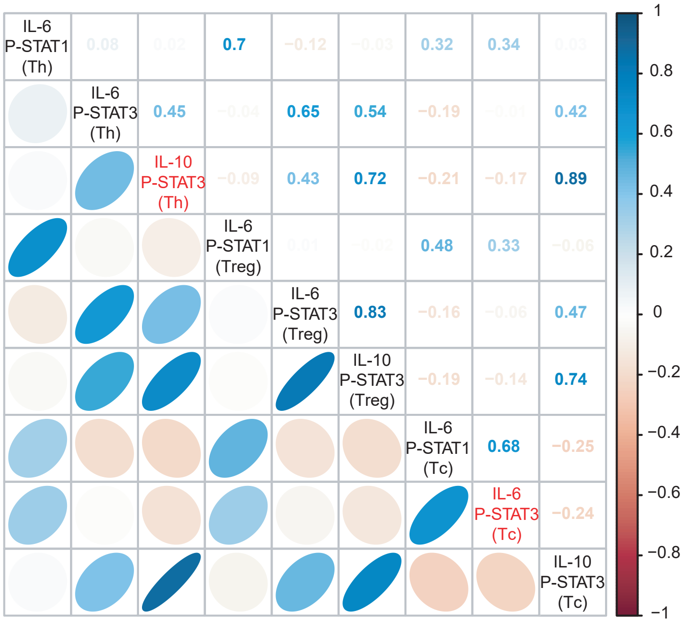

Figure S1. Feature selection and correlation of features. **A.** Feature selection based on MeanDecreaseGini. From the highest, two values were selected. **B.** The correlation plot for nine CIPS signatures. The two selected features (red), IL-6 induced P-STAT3 on CD8+ T (Tc) and IL-10 induced P-STAT3 on CD4+ T (Th), showed no correlation, indicating that the two features are independent to each other.

Figure S1
